# Exploring acupuncture as a therapeutic approach for tic disorders: a review of current understanding and potential benefits

**DOI:** 10.3389/fneur.2025.1447818

**Published:** 2025-03-14

**Authors:** Haoyang Liu, Chunping Wang, Hongbin Zhang, Mohammad J. Rezaei

**Affiliations:** ^1^School of Pharmacy, Liaoning University of Traditional Chinese Medicine, Dalian, Liaoning, China; ^2^Shouguang Hospital of T.C.M. Emergency Department, Shouguang, Shandong, China; ^3^School of Medicine, Tehran University of Medical Sciences, Tehran, Iran

**Keywords:** natural compound, acupuncture, tic disorders, therapy, bio-active compound

## Abstract

Tic disorders (TD) refer to a condition where individuals experience recurring motor movements (e.g., eye blinking) and/or vocalizations (e.g., throat clearing). These disorders vary in terms of duration, cause, and manifestation of symptoms. Tourette’s syndrome (TS) involves the presence of ongoing motor and vocal tics for a minimum of 1 year, with fluctuating intensity. Persistent chronic motor or vocal tic disorder is characterized by either motor or vocal tics (not both) present for at least 1 year. Provisional TD presents with either motor or vocal tics (not both) that have been present for less than 12 months. Though medications like Aripiprazole and dopamine receptor blockers are frequently prescribed, their potential unwanted consequences increase, may result in low adherence. In an effort to improve and broaden the care available for children diagnosed with TD, alternative methods such as acupuncture are being investigated and considered. Acupuncture is a method of traditional Chinese medicine that includes the placement of thin needles into particular areas of the body in order to correct any disruptions or irregularities. Research has demonstrated that acupuncture can help regulate abnormal brain function and relieve tic symptoms in individuals with TD. Additional studies are required to fully evaluate the usefulness of complementary treatments in addressing TD in young individuals, despite its common usage. Herein, we summarized the therapeutic effects of acupuncture in the treatment of TD.

## 1 Introduction

Tic disorders (TD) are common neurological issues that are prevalent among young people (1). The Diagnostic and Statistical Manual of Mental Disorders, Fifth Edition (DSM-5) classifies tic disorders into three categories: Tourette syndrome (TS), persistent motor or vocal tic disorder, and provisional tic disorder (2–5). These disorders involve repeated, abrupt, and irregular vocalizations or muscular actions and affect about 6 to 20% of children globally (6).

At present, TD are often treated with the use of haloperidol, risperidone, Aripiprazole, tiapride, and clonidine. Due to the fact that TD are commonly long-lasting, it is usually necessary to take medication for an extended period. The extended use of medication may lead to several adverse effects on patients, including weight gain, drug-induced movement disorders, high levels of prolactin, fatigue, and alterations in heart rate, blood pressure, and electrocardiograms (7). For over two millennia, China has utilized acupuncture as a form of treatment (8). This method of treatment offers numerous benefits when compared to medication-based therapy for certain illnesses, with regards to its safety, efficacy, convenience, and reduced risk of adverse reactions. Hence, acupuncture is recognized as a legitimate and efficient form of complementary or alternative treatment, often employed for the prevention and management of neurological and psychological disorders (9, 10). The effectiveness of this treatment approach for TD needs to be determined through empirical evidence (11). Based on the information we have; some limited studies have shown that acupuncture can have positive results in treating TD in children. In fact, it may even be more effective than using medicine alone. However, given the small number of participants and inadequate study design, further research is necessary to confirm these findings (12). According to ancient Chinese beliefs of acupuncture, good health is attained through the constant and unobstructed circulation of Qi (Qi in traditional Chinese medicine (TCM) refers to the vital energy or life force that flows through the body). It is essential for maintaining health, and its balance ensures proper functioning of the body. Qi moves through pathways called meridians, and disruptions in its flow can cause illness. Treatments like acupuncture and herbal medicine aim to restore and balance Qi for overall well-being (13). The feeling of Deqi [described as a distinct, sometimes intense, sensation that signals the effective activation of the meridian or acupuncture point being treated (14)] that occurs during acupuncture is the primary element that determines the overall effectiveness of the treatment. Recent research using advanced brain imaging techniques has brought forth fresh evidence supporting the therapeutic effect of Deqi during acupuncture for patients with ischemic stroke, depression, and potentially even for children with TS (15, 16). Herein, we summarized the therapeutic effects of acupuncture in the treatment of TD.

## 2 Tic disorders pathophysiology

In the past, TD was perceived as an enigmatic condition and its exact origins were largely unclear (17). Over time, research has shown that TD includes various neurodevelopmental disorders, as seen through both clinical observation and fundamental studies (18). TD’s origin can be linked to a combination of genetic, immune, mental, and external elements. The connection between the root causes and noticeable indications probably stems from the absence of control in the pathways connecting the brain’s outer layer, striatum, thalamus, and returning to the outer layer (19–22). The tics and related symptoms are thought to be caused by an uneven distribution of inhibitory and excitatory signals in these circuits. This can be attributed to factors such as heightened levels of striatal dopamine or increased sensitivity of dopamine receptors, resulting in the manifestation of tic symptoms (19, 23, 24). Various irregularities in neurochemicals and neurotransmitters have been associated with TD/TS, particularly in the pathways involving dopamine, adrenaline, Gamma-Aminobutyric Acid (GABA), and glutamate (19, 22, 25). Newer research has pinpointed a link between TD/TS and pathways involving histamine and endogenous cannabinoids through the use of genetic, pharmacological, and brain imaging investigations (26–29).

In addition, recent comprehensive research in genetics, brain imaging, and brain activity has indicated that TD/TS, whether accompanied by Attention-Deficit/Hyperactivity Disorder (ADHD) and Obsessive-Compulsive Disorder (OCD) or not, do not stem from distinct disorders, but rather arise from similar developmental problems in the cortical-striatal-thalamo-cortical circuits of the brain. These circuits have a function in triggering, choosing, carrying out, acquiring, and strengthening planned actions, ideas, conduct, and emotions (19, 20, 30, 31). Tics could be due to disruptions in the sensorimotor and oculomotor pathways, whereas Obsessive-Compulsive Behaviors (OCB)/ OCD manifestations may stem from disruptions in the anterior cingulate and lateral orbitofrontal pathways. Similarly, imbalances in the dorsolateral prefrontal circuit may be associated with ADHD symptoms (19, 20, 30).

Previous studies have suggested that the presence of TD/TS is heavily influenced by genetic factors, with an estimated heritability of 0.77. However, the exact gene responsible for causing TS remains unidentified (32, 33). The biggest genome-wide association and family co-segregation studies to date involved 4,819 individuals with TS and 9,488 control subjects. The findings showed that there was only one notable area on chromosome 13 [Fms-like Tyrosine Kinase 3 (FLT3)] identified by the marker rs2504235, which had an odds ratio of 1.16. Moreover, the research revealed that utilizing polygenic risk scores could effectively forecast the occurrence of TS and tic spectrum disorders within a population-based group (34). Furthermore, a research study that examined information from 232,964 individuals diagnosed with psychiatric disorders and 494,162 individuals without any known disorders, identified 109 regions in the genetic code that were linked to at least two disorders. Additionally, these regions were also linked to four or more disorders, including ADHD, OCD, and TS. These areas are primarily located within genes that demonstrate significant activity in the brain and have a critical role in processes related to the development of the nervous system (35). These results suggest that TD/TS may have a complex genetic basis and may share common genetic origins, underlying mechanisms, and neural pathways with its comorbidities.

## 3 Treatment of tic disorders

**Figure 1:**
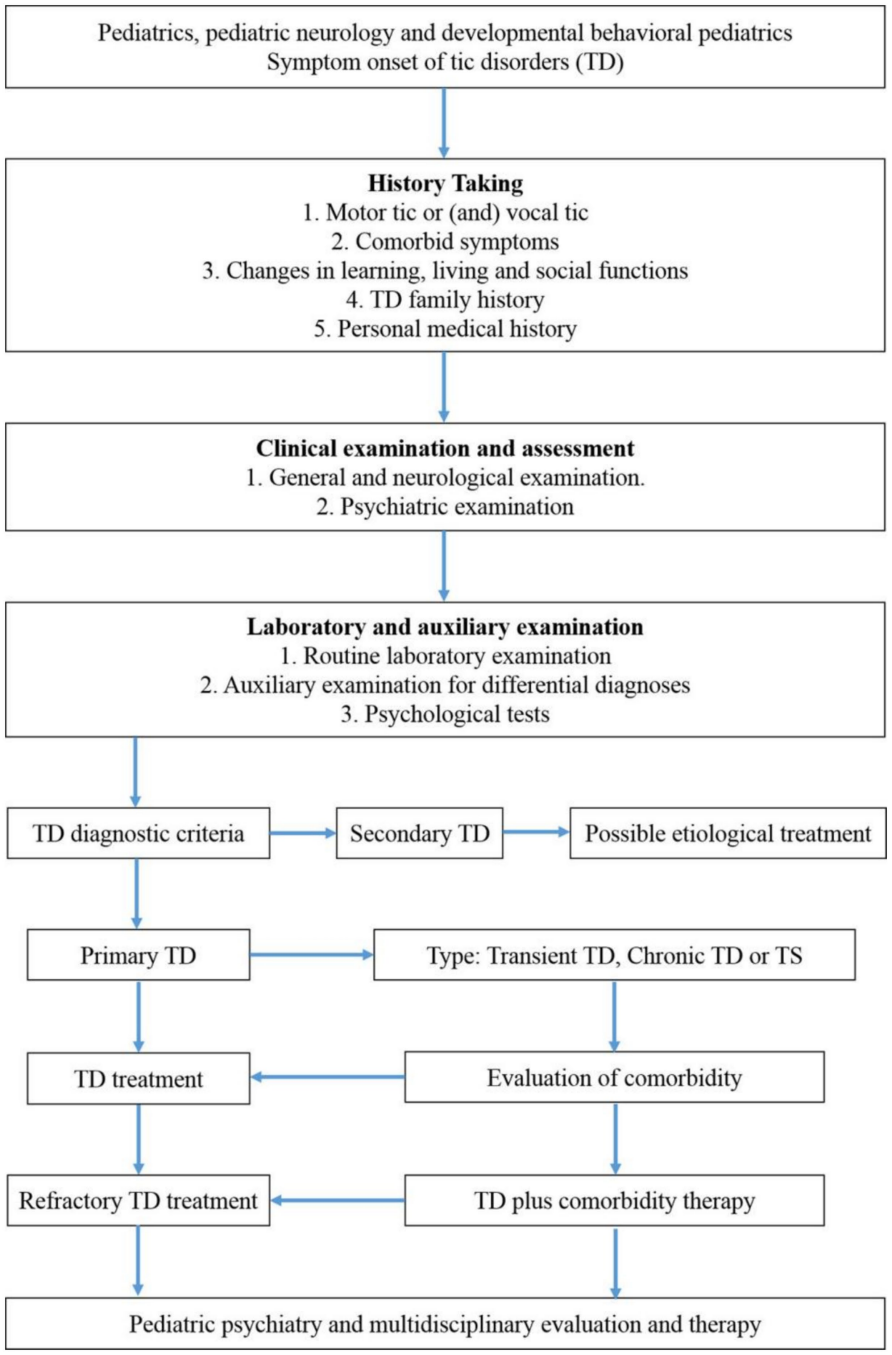
A simplified diagnostic and treatment roadmap of TD based on current guidelines in China.

Proper diagnosis and treatment of TD is important as it could be associated with other comorbidities and only its comorbidities such as ADHD being treated (36). Figure 1 provide a comprehensive overview about diagnosis and treatment of tic disorder based on current recommended guidelines in China (36).

In the last decade, multiple experts and clinical evidence have led to the adoption of consensus treatment recommendations for TD/TS in Western countries. In order to create a successful treatment plan for tics, a thorough evaluation of the tics and any accompanying psychological, social, or behavioral concerns must be conducted. It is essential to determine how these issues affect the individual and their daily functioning. In some cases, children and teenagers with tics may not need any intervention or therapy unless their tics significantly disrupt their daily routines or school responsibilities (37). In China, the accessibility of cognitive-behavioral therapy (CBT) and medication treatments may differ, and clinician perspectives may also influence the implementation of clinical guidelines in various locations and situations (38). Thus, it is crucial to make treatment decisions considering the individual requirements and available resources of the patient, along with the skills of the healthcare provider and guidance from reputable associations. Before starting treatment, it is crucial to identify the most significant symptoms that impact a patient’s daily life, studies, or social interactions. While tics are typically the primary focus of treatment, for some children, other comorbid symptoms like hyperactivity, impulsiveness, and obsessive-compulsive behaviors may be the main target (39, 40). For children experiencing mild TD, the option of providing medical education and psychological support may suffice initially, with a recommended period of observation and regular check-ins (41). The approach to treating moderate to severe TD involves utilizing non-medical methods initially, and incorporating behavioral therapy alongside medication if necessary (42–44). Further interventions such as deep brain stimulation could be used if necessary but these interventions and their tolerability are still in debate (45). In spite of this, comprehensive medical training and emotional assistance must be offered throughout the entire period of treatment (36).

European guideline for treatment of TD suggested that dopamine blocking agents, such as Aripiprazole could be considered as a first line treatment due to its minimal side effects compared to other antipsychotics. In addition to Aripiprazole, tiapride and risperidone could be added to the therapeutic regimen in the case of coexisting ADHD (46).

Vesicular Monoamine Transporter 2 (VMAT2) inhibitors prevent the activity of VMAT2, the protein responsible for packaging monoamines, like dopamine, into small sacs inside the nerve cells to be released into the space between the cells (47). VMAT2 inhibitors cause a reduction in dopamine release in the striatal area, leading to a depletion of this chemical messenger. Some examples of medications in this group are tetrabenazine, deutetrabenazine, and valbenazine. The FDA has approved deutetrabenazine for controlling symptoms of Huntington’s chorea and tardive dyskinesia, and valbenazine specifically for treating tardive dyskinesia. Off-label, these inhibitors have proven to be effective in treating various hyperkinetic movement disorders, including tics (47, 48).

In a study of 23 individuals aged 12–18 with TS, a mean daily dosage of 32.1 mg of deutetrabenazine (with a maximum of 36 mg per day) was administered for 8 weeks in an uncontrolled trial (49).

Past studies have examined the potential of utilizing marijuana as a treatment for TS. A study involving 12 adult patients with TS found that those who were given delta 9-tetrahydrocannabinol showed significant decreases in tics and obsessive-compulsive behavior 3–4 h after treatment, in comparison to those who were administered a placebo. This was concluded by using the TS symptom list, which revealed a notable disparity in variation compared to the initial state (50). A distinct but minimal shift towards tic enhancement was noticed in the treatment cohort according to the Shapiro TS severity gauge, the global scale for TS, and the Yale Global Tic Severity Scale (YGTSS). The examiner also observed a connection between the improvement of tics and the measurement of tetrahydrocannabinol (THC) levels in the serum (50). In a later 6-week Randomized Double-Blind Placebo-Controlled Trial (RDBPCT), which had an additional 24 participants, it was observed that the THC-treated group showed a similar level of improvement to the placebo group by the end of the trial. Nevertheless, this result did not attain statistical significance (51).

Several studies have demonstrated the effectiveness of drugs known as alpha-2 agonists, such as clonidine and guanfacine, in treating TS, particularly in children and adolescents (52–57). Due to their relatively low risk of side effects, these medications are frequently recommended by specialists in children’s health or mental health as a primary treatment for patients with mild tics. They have also been researched in individuals who have slight ADHD or impulsive behavior disorder. Multiple experiments comparing these medications to a control group have demonstrated the efficacy of clonidine. In a 16-week trial with 136 children diagnosed with ADHD and chronic TD, participants were randomly given either clonidine, methylphenidate, a combination of both, or a placebo (54). The YGTSS was used to assess tics, with scores ranging from 0 to 100 as the main outcome measure. The results from all the treatment groups outperformed the placebo group in reducing tic severity. The most notable enhancement was observed in the clonidine-methylphenidate combination group, where a decrease of 11.2 (98.3% confidence interval − 0.1, 22.5) was reported. Similarly, the clonidine-only group also showed a considerable reduction of 10.9 (98.3% confidence interval 2.1, 19.7). Contrary to popular belief, it was found that the use of central nervous system (CNS) stimulants did not worsen tics, as proven by the study which showed no negative impact of methylphenidate. Different research with 437 individuals who had tics or TS also proved that clonidine is an effective treatment for reducing tics (52). Participants randomly assigned to receive a clonidine patch for treatment showed a significant reduction in tics, as measured by the YGTSS, compared to those who were given a placebo. Various investigations have determined that topiramate is a successful remedy for tics linked with TS (58, 59). For a period of 10 weeks, 29 individuals with a diagnosis of TS, aged between 7 and 65 years, were randomly divided into two groups to receive either topiramate or a placebo in an initial randomized double-blind trial (58). The topiramate group saw a larger reduction in YGTSS-TTS compared to the placebo group, with a difference of 14.29 ± 10.47 points. The placebo group only showed an improvement of 5.0 ± 9.88 points. While the improvement in premonitory urges was noted in the topiramate group, it was not measured quantitatively.

A study that analyzed 14 trials with 1,003 participants between 7 and 17 years old, looked at the effectiveness of topiramate for treating tics compared to traditional treatment methods (60). In every experiment, a medication was utilized as a standard for contrast; 12 of the experiments utilized haloperidol while 2 utilized tiapride. In three of the trials, the YGTSS was utilized to evaluate results and it was revealed that there was a significant enhancement in tics when topiramate was administered. The average reduction in the overall YGTSS score was 7.74 points, with a 95% certainty ranging from 10.49 to 4.99. In the other trials, topiramate did not show any superiority when compared to either haloperidol or tiapride (60).

Although currently available medications that inhibit dopamine receptors primarily function by decreasing D2 receptor activity, targeting D1 receptors may also have potential benefits. Ecopipam, a D1 receptor blocker, was originally created as a potential treatment for antipsychotic medication in the 1980s, but it was unsuccessful in clinical trials for schizophrenia (61, 62). Yet, it has demonstrated potential in addressing tics. In one unregulated investigation, a group of 18 subjects (with an average age of 36.2 years) received a 100 mg dosage of ecopipam for a period of 8 weeks (63). After the study was completed, there was a significant decrease in YGTSS-TTS as compared to the initial measurement. The numbers decreased from 30.6 ± 8.8 to 25.3 ± 9.2. Additionally, there was a noticeable enhancement in the YGTSS impairment rate, with a decrease from 29.7 ± 10.9 to 22.8 ± 13.7. The most commonly reported negative effects included drowsiness, exhaustion, trouble sleeping, and feelings of nervousness, migraines, and involuntary muscle contractions.

## 4 Natural compounds and neuropsychiatric disorders

Neuropsychiatric disorders refer to mental and brain disorders frequently linked to dysfunction in the brain (64, 65). Numerous scientists utilize advantageous therapies with minimal adverse reactions to address the needs of these individuals. Therefore, the appropriate form of treatment is based on the assortment of ailments the individual is experiencing (66). Individuals who have suffered a brain injury tend to have a heightened sensitivity to the adverse effects of pharmacological drugs as compared to those without any injury. As a result, it is imperative for doctors to exercise caution when selecting the most suitable medication, dosage, and duration of treatment for these patients (67–69). Various research studies using animal subjects have demonstrated that certain medicinal substances, including haloperidol, benzodiazepines, and clonidines, can hinder the healing of damaged neurons and ultimately disturb the usual functions of the brain (70). Existing drugs for neuropsychiatric disorders primarily focus on managing the symptoms of the disease. As a result, there is a pressing need to create treatments that can slow down, prevent, or even reverse the advancement of the disorder (71).

Clinical trials utilize antioxidants to disrupt the advancement of diseases, however the outcomes are inadequate. The majority of these antioxidants indiscriminately aim at neuroprotective pathways. As a result, additional research is required to uncover novel potential substances that can restore redox equilibrium while also decreasing neuronal harm (72). Modernly, the utilization of herbal remedies as a substitute for traditional medicine is gaining popularity due to their low risk of adverse effects on the body (73).

Certain plants can alter one’s emotional state through their impact on the transmission of monoamine neurotransmitters. This is similar to how *Hypericum perforatum*, also known as St. John’s Wort, affects the brain. These plants also have an influence on other systems such as GABA, opioids, and cannabinoids (74, 75). For instance, certain alkaloids that resemble membranes and are found in plants such as *Narcissus* (*Amaryllidaceae*) and *Sceletium* possess promising anti-depressant effects (74, 76). Narcissus is a plant that contains neuroactive compounds, such as galantamine, which have been utilized in therapies for Alzheimer’s disease (77). Alkaloids with similar properties to mesembrine showed promise in treating mood disorders by acting as selective serotonin reuptake inhibitors (SSRIs) (78). Furthermore, it has been demonstrated that mesembrine alkaloids possess phosphodiesterase-4 (PDE-4) inhibitory properties. This is accomplished by changing the amount of cyclic AMP (cAMP) and causing the production of Brain-Derived Neurotrophic Factor (BDNF) mRNA, leading to a positive impact on those with Major Depressive Disorder (MDD) (79).

According to experts, Curcumin, a natural compound present in *Curcuma longa*, is strongly suggested for managing MDD (80). Several writers have asserted that curcumin has a similar effect on anxiety-ridden mice as imipramine in the forced swim test (FST), as it can regulate various neurotransmitter systems (81, 82). In a separate research, the manipulation of the serotonin system through the stimulation of the cAMP pathway using curcumin was validated (83). Curcumin’s ability to function as an antidepressant is partially due to the involvement of glutamate receptors, which work to inhibit presynaptic voltage-gated calcium channels (83). A study found that curcumin decreased glutamate release and increased the antidepressant effects of fluoxetine (83–87). The reports showed that apigenin, a type of bioflavonoid, had a strong effect on reducing immobility and promoting neurotransmitter production in behavioral tests conducted on mice (88). Furthermore, haloperidol inhibited the antidepressant effects of apigenin (89). At a dose of 20 mg/kg given orally, its antidepressant properties were observed in rats through inhibiting interleukin 1β and stimulating the NOD-, LRR- and pyrin domain-containing protein 3 (NLRP3) inflammasome in the brain (90). Amentoflavone, a compound derived from bioflavonoids,was found to hinder the binding of flumazenil to GABA receptors in the brains of rats (91–95). Some researchers observed that Amentoflavone given orally in the forced swim test had a stronger effect compared to imipramine (93).

**Figure 2:**
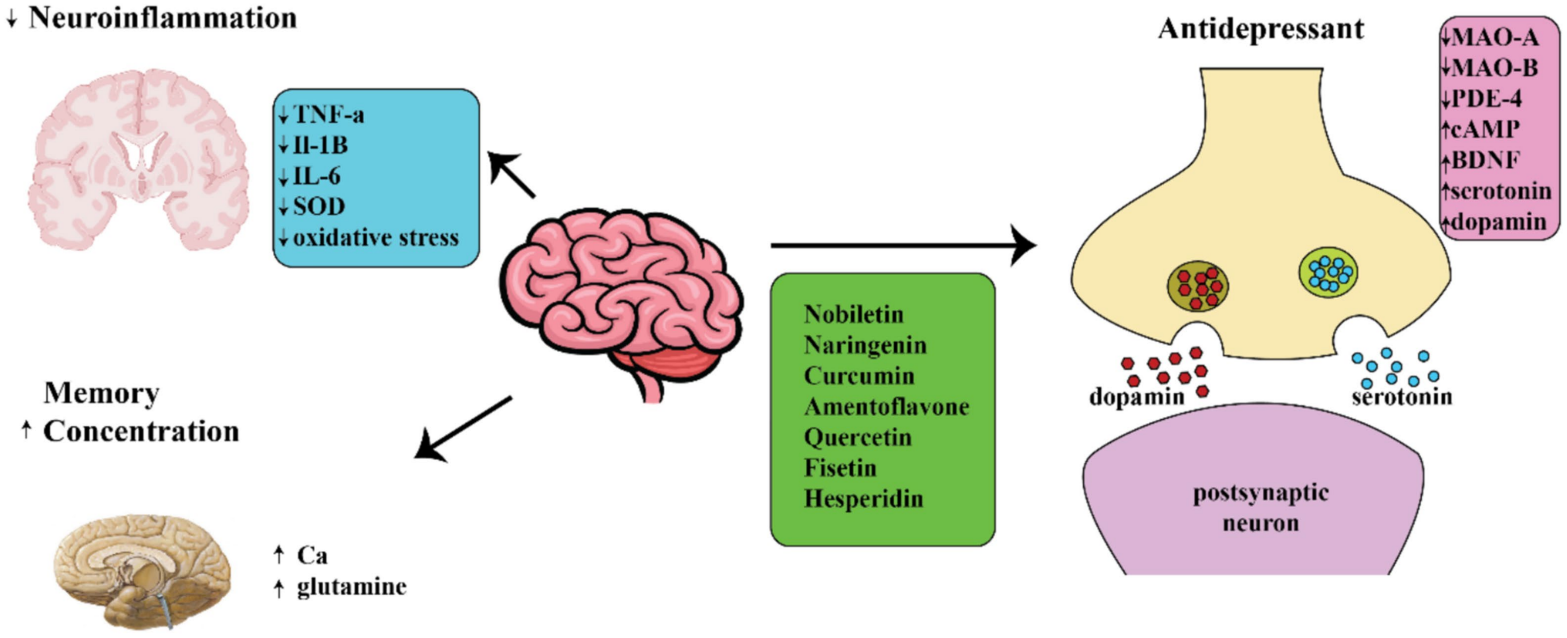
Diagram showing different ways in which natural compounds may act on neuropsychiatric disorders.

In previous research, it has been shown that chlorogenic acid, a compound present in coffee known as a polyphenol, may have the ability to improve mood in individuals with certain conditions (96). The opioidergic pathway was postulated as the means by which chlorogenic acid exerts its antidepressant effects (97–99). One could also decrease instances of neuroinflammation and oxidative stress (100). Ferulic acid has been shown to produce an anti-immobility response in models of behavioral despair, such as the FST and TST. It can also be used as a beneficial supplement for treating depression in individuals with epilepsy (101, 102). Several research studies have indicated that the quercetin bioflavonoid has the ability to act as an antidepressant by suppressing the function of MAO in the brain and balancing the levels of copine 6 and the triggering receptors expressed on myeloid cell ½ (TREM1/2), which are associated with the BDNF factor (Figure 2) (103–107). Moreover, quercetin exhibited anti-depressant properties in streptozotocin-induced diabetic mice, comparable to those of fluoxetine and imipramine (108, 109). In certain research, it was observed that Quercetin was able to prevent the deterioration of serotonin neurotransmitters in the mitochondria of mouse brains (110). Hesperidin, another compound, decreased the length of the period of inactivity in the animal model for locomotor activity (111).

## 5 Nature compounds and tic disorders

The investigation into natural substances for the treatment of TD is a rapidly developing area, motivated by the demand for therapies that are potent, have minimal adverse reactions, and target both the neurological and psychological components of the condition. Studies on substances like magnesium and omega-3 fatty acids have shown great potential. Specifically, magnesium is vital for nerve signal transmission and muscle movement, and inadequate levels of this mineral have been associated with heightened nerve activity. Using magnesium as a supplement could potentially assist in regulating the excitability that is essential in managing the involuntary tics associated with TD. Additionally, omega-3 fatty acids play a role in maintaining cell membrane flexibility and can produce anti-inflammatory compounds, potentially aiding in the decrease of neuroinflammation that worsens TD.

Further examining the capabilities of plant-derived compounds, *Ginkgo Biloba* and Ashwagandha have been recognized for their ability to protect the nervous system and promote mental well-being. This is particularly significant as ADHD and anxiety frequently accompany TD, potentially due to its ability to dilate blood vessels and reduce harmful oxidative substances in the brain. The impact of Ashwagandha extends beyond managing tics and also promotes cognitive health, making it a valuable treatment for ADHD symptoms commonly seen in individuals with TD. Its adaptogenic characteristics help regulate the body’s reaction to stress, which is crucial in light of the fact that stress and anxiety can exacerbate tic symptoms. This herb serves as a natural means of bolstering the body’s ability to handle stress, thus effectively reducing the frequency of tics.

By integrating these organic components into medical treatment, it has the potential to revolutionize the way TD are traditionally treated. By promoting a comprehensive treatment method, medical professionals can not only target the specific underlying neurological issues of TD, but also enhance the overall well-being of patients by alleviating the mental anguish linked to the condition. This methodology aligns with the current trends in healthcare, which prioritize a holistic and personalized approach that caters to the distinctive biological and psychological aspects of each individual patient. It additionally corresponds with patient-centered care approaches, which focus on addressing the patient as a whole, taking into account their physical, emotional, and social requirements.

## 6 Overview of acupuncture techniques

Acupuncture needling and moxibustion are fundamental techniques essential for effective clinical treatment in acupuncture. These techniques are of great importance in the field of acupuncture, serving as a crucial connection between the theoretical foundations and practical applications in traditional Chinese medicine. Both acupuncture needling and moxibustion are external methods used to treat various conditions, with the former utilizing a variety of needles and techniques to stimulate specific areas and regulate the body’s functions through channel Qi stimulation, and the latter employing substances like wormwood to warm the body surface and produce therapeutic effects (112). There is a plethora of methods for acupuncture needling and moxa techniques, each with varying approaches to needle placement, levels of stimulation, and areas of focus. The execution of each technique in clinical settings involves particular steps and procedures that differ from one another. Proper implementation is crucial not only for the safety and effectiveness of the treatment, but also for its success in achieving desired outcomes. Mastering the art of acupuncture techniques requires dedication, as it is a continuous process of learning and refinement. In clinical practice, it is imperative for acupuncturists to carefully select the most suitable techniques based on the unique features of the patient’s ailment and other relevant factors. This demands a comprehensive understanding of the theory behind acupuncture and proficiency in its practical application, acquired through extensive theoretical education and hands-on training (113).

Scalp acupuncture is a frequently utilized method for treating neurological disorders such as stroke rehabilitation, Parkinson’s disease (PD), and multiple sclerosis (MS). This procedure involves the insertion of needles into precise regions of the scalp that correspond to specific areas of the brain anatomically. It is believed to activate the underlying brain regions and has shown positive results in neuro-rehabilitation by targeting zones that correspond to the brain regions responsible for motor function and sensation. Experiments and studies, including those evaluated in Cho et al.’s research, have demonstrated the potential of scalp acupuncture in improving recovery after a stroke (114).

Body Acupuncture is a widely practiced form of acupuncture that involves the insertion of needles into predetermined areas of the body. This method is commonly employed to address a range of ailments such as pain, gastrointestinal issues, and psychological disorders like anxiety and depression. Body acupuncture utilizes points located along the meridians of the body, each linked to specific organs and health conditions, making it a versatile approach. In a notable study conducted by Lee et al., the effectiveness of body acupuncture in alleviating chronic pain is extensively evaluated (115).

Auricular acupuncture is a therapeutic technique that targets the ear as a microsystem mirroring the entire body. Often utilized for addiction rehabilitation, alleviating pain, and inducing calmness, it stimulates particular points on the ear which can impact a range of ailments. According to Smith et al., its effectiveness has been shown in diminishing anxiety in individuals before surgery, highlighting its potential for acute treatments (116).

The Qihuang Needle (QHN) technique is a method that is not widely recognized but holds great historical importance. QHN Therapy is a modern acupuncture method based on traditional Chinese medicine principles. Named after Qi Huang, it uses specialized needles and precise techniques to balance Qi and treat various conditions, focusing on holistic healing with minimal discomfort (14, 117).

It has been traditionally used in Chinese medicine to enhance the body’s natural defensive abilities and remove harmful agents. Zhou et al.’s exploration of the QHN technique combines traditional acupuncture practices with modern knowledge of the immune system, providing a comprehensive approach to managing autoimmune disorders (112).

Electroacupuncture is a form of acupuncture that involves the insertion of two needles into specific points on the body and the use of a mild electric current. This technique is based on traditional acupuncture methods and is particularly useful for managing pain and treating neurological disorders. Once the needles are inserted, they are connected to an electrical device that delivers ongoing pulses of electricity. The frequency and strength of the pulses can be altered to suit the needs of the patient. This method is thought to increase the flow of qi more effectively than manual stimulation, which may result in improved results for conditions such as chronic pain, paralysis, or inflammation (118).

Moxibustion is a treatment method that involves using a heat source, created by burning dried plant materials known as “moxa,” near or on specific points on the skin called acupuncture points. By doing so, the body’s flow of qi and blood is influenced. This technique is often combined with traditional acupuncture to enhance its therapeutic benefits. The moxa is typically made from the mugwort herb and can take the form of cones, sticks, or be directly placed on an acupuncture needle. The heat produced from the moxa is applied either in close proximity to the skin or on top of the needle, without causing burns. Moxibustion is especially beneficial for conditions characterized by coldness and stagnation, such as chronic pain, where the warmth can promote better blood flow and relieve symptoms (119).

Tui Na is a type of traditional Chinese therapy that involves manipulation and stimulation of the body. In addition to using acupuncture and moxibustion, Tui Na utilizes various hand techniques to massage the muscles and tendons and apply pressure on specific points to influence the flow of Qi. Some common techniques used include brushing, kneading, rolling/pressing, and rubbing the areas between joints known as the eight gates. The goal of Tui Na is to stimulate and balance the body’s defensive Qi, promoting the flow of energy within the meridians and muscles. It is not limited to addressing physical ailments, but also has the ability to enhance energy and promote relaxation, making it a versatile treatment for both physical and emotional issues (120).

## 7 Acupuncture and neurological disorders

Acupuncture is an established and popular alternative medicine that has proven to be effective in treating various neurological conditions, especially those related to degeneration of the nervous system (121, 122). The aim of acupuncture therapy is to alleviate symptoms by inserting a needle into particular acupoints on the skin’s surface and using manual manipulation or electrical stimulation (123). Acupuncture can effectively treat different neurological disorders by stimulating the peripheral nerves to promote healing and recovery (124–126).

Acupuncture is a highly regarded therapy among individuals suffering from mild to moderate Alzheimer’s disease (AD). In a significant investigation, a total of 87 individuals with mild to moderate Alzheimer’s disease were subjected to three different treatments for a period of 12 weeks: individual treatment, three acupuncture sessions per week, or daily intake of donepezil. In 28 weeks, the acupuncture group had notably lower AD assessment scale-cognitive subscale (ADAS-cog) scores than the donepezil group, according to the findings. Furthermore, there was a significant decrease in the mean scores of Clinician’s Interviews Based Impression of Change-Plus (CIBIC-Plus) among the acupuncture group at both week 10 and 28. Positive changes were observed in cognitive abilities after the 12-week course of acupuncture treatment (127). In another small experiment, eight participants with mild to moderate Alzheimer’s disease received acupuncture treatment. Each individual was given medical care for a duration of 30 days, consisting of a 7-day course of treatment followed by a 3-day rest period. The cognitive abilities of the individuals were assessed using speech-based orientation, physical coordination, and the overall result from the MMSE (Mini-Mental State Examinatio). The findings revealed considerable enhancement in cognitive ability and noteworthy progress in clinical symptoms based on the Traditional Chinese Medicine Symptoms Checklist for AD (128, 129).

Acupuncture is shown to greatly enhance the nocturnal sleep experience of those with Parkinson’s disease. Sleep disorders, commonly characterized by inadequate sleep and post-sleep tiredness, are widespread among this population (130–132). Disturbances in sleep have been linked to a decline in the body’s effectiveness in eliminating metabolic waste and an elevation in oxidative stress. As a result, an excess of *α*-synuclein accumulation occurs which leads to neuronal loss. This procedure ultimately influences the progress and growth of Parkinson’s illness (133, 134). Acupuncture has been found to effectively enhance sleep quality in mouse models of Parkinson’s disease induced by 1-Methyl-4-Phenyl-1,2,3,6-Tetrahydropyridine (MPTP). This is achieved through the regulation of Serum- and Glucocorticoid-Regulated Kinase 1 (SGK1) levels and decreasing the buildup of α-synuclein (135).

Acupuncture is a widely employed technique for relieving both sudden and long-lasting discomfort (136, 137). Through analysis of 31 randomized controlled trials, Kang et al. highlight a significant concern related to tension-type headaches (TTH) (138). It was determined that acupuncture could potentially be a harmless and beneficial remedy for those with TTH. In a randomized controlled experiment, Liu et al. investigated the impact of consuming caffeinated beverages on the pain-relieving benefits of electroacupuncture. This study provides possible reasons for the differences in the success of acupuncture practice between Western and Eastern cultures (139). Based on Lee et al.’s findings, current evidence suggests that acupuncture shows potential as a therapeutic approach for controlling symptoms of an overactive bladder (OAB). In comparison to sham acupuncture, acupuncture seemed to have a more significant effect in decreasing symptoms of overactive bladder. Furthermore, acupuncture demonstrated comparable efficacy to traditional medication in enhancing symptoms associated with OAB (140). Huang Y. and colleagues conducted a comprehensive evaluation of scholarly literature regarding the use of acupuncture in the treatment of spinal cord injury (SCI). The examination showed that China and the US were the main generators of research in this particular area. In addition, the scientists predicted a rise in the study of electroacupuncture in promoting nerve recovery and regeneration after spinal cord injury (141). Zhang and colleagues employed Functional magnetic resonance imaging or functional MRI (fMRI) to investigate the influence of acupuncture on brain activity and neural mechanisms. According to their findings, acupuncture has the ability to decrease the severity of subjective tinnitus by decreasing the functional connections of the amygdala (142). The authors Huang H. et al. conducted a meta-analysis to examine the impact of acupuncture on brain activity. The results showed that stimulating the Stomach 36 (ST36) acupoint caused a rise in activity in the Inferior frontal gyrus, right hemisphere (IFG.R), Superior temporal gyrus, left hemisphere (STG.L), and Middle cingulate gyrus, right hemisphere (MCG.R) regions of the brain (143). In two other studies (143, 144), examined the most effective methods of using acupuncture to treat TD and mild cognitive impairment. These investigations will offer valuable materials for scientists exploring the choice of acupuncture points and the integration of fMRI methods in acupuncture therapy. Furthermore, aside from the aforementioned findings on BP, there were also four more instances reported, consisting of a 50-year-old female experiencing hemifacial spasm and temporomandibular joint pain, a man and woman with impaired sense of smell after contracting COVID-19, a woman diagnosed with Guillain-Barré syndrome, and a child in a persistent vegetative state due to herpes simplex virus encephalitis (145–147). All of these people achieved positive results in their healing after receiving acupuncture treatment. Epilepsy is a highly prevalent and severe neurological disorder that causes seizures. The outlook for those affected is typically grim, as it is a serious condition with significant neurological symptoms (148). The Acupuncture and Moxibustion Scientific Research Center (AMSRC) scientists propose that administering kainic acid (KA) through intracerebral injection in the hippocampus or amygdala of rats can serve as a simulation of temporal lobe epilepsy. This is due to the similar histopathological alterations observed in individuals with epilepsy (148). Kim et al. (149) showed that acupuncture was successful in preventing seizures and protecting hippocampal cells from KA-induced cell death by enhancing the production of glutamate decarboxylase-67. Through the use of acupuncture at HT8, the intensity of KA-induced seizures and the frequency of neural cell death were lessened. The research also noted a decline in the presence of c-Fos and c-Jun, which are triggered by KA and indicate increased activity in neurons, in the hippocampus using methods such as immunohistochemistry and Western blotting. Additional studies revealed that the use of acupuncture on HT8 effectively suppressed the activation of microglial cells and reduced the levels of inflammatory substances, such as interleukin (IL)-1β, in the hippocampus (150). According to the findings of this research, acupuncture has multiple positive impacts on individuals experiencing epileptic episodes. These include controlling specific gene functions and decreasing inflammation. One potential benefit of acupuncture is its ability to enhance hippocampal cell proliferation and decrease hippocampal cell apoptosis, which may help counter the consequences of decreased blood flow. The application of acupuncture on particular acupoints like ST36 and LI4 may have a significant impact on controlling cellular growth and decay following ischemic damage (151). Research was conducted to investigate the impact of acupuncture on ST36 acupoint in rats with intracerebral hemorrhage. The experiment focused on observing the changes in Fos expression and cell proliferation in the dentate gyrus area (152). Acupuncture treatment may aid in the recovery of stroke patients by lessening cell damage and stimulating cell growth caused by inadequate blood flow.

## 8 Acupuncture and tic disorders

Recent research has consistently demonstrated that acupuncture is a highly beneficial method for managing TD (153). Several forms of acupuncture have been found to have positive effects on total clinical effectiveness, adverse events, and recurrence, specifically electroacupuncture and scalp acupuncture. TD are often treated with various forms of Traditional Chinese medicine, such as acupuncture, electroacupuncture, scalp acupuncture, and auricular acupuncture (154). However, these techniques come with certain drawbacks, including the need for the needle to be left in place for extended periods, difficulties in controlling the electric current, and the requirement for the child to initiate the treatment. Moreover, the majority of patients are school-aged children who are under significant academic stress, which calls for short-term courses of treatment with adaptable schedules.

QHN therapy provides advantages as opposed to traditional acupuncture (155). To start, the discomfort felt when using the QH needle is very minimal. Furthermore, the needle does not have to remain inserted in the skin for a prolonged duration. Choosing acupoints is uncomplicated and typically only requires up to five points per session. Lastly, QH acupuncture yields faster results, particularly for stubborn pain, compared to traditional Chinese acupuncture.

Numerous research studies have been conducted to assess the efficacy of QHN therapy for treating various medical conditions such as low back pain, acute gouty arthritis, and Parkinson’s disease (117, 155, 156). QHN therapy has been utilized in the field of childhood neurological and psychiatric disorders, such as TD and spastic hemiplegic cerebral palsy (155). It seems that QHN therapy may offer potential as a potential method for treating TD. Further investigation is required to examine its efficacy and underlying mechanisms. Tics are caused by disruptions in the transmission of signals between neurons in the cortico-basal ganglia network (20). Changing the composition of intestinal microbes may play a critical role in the emergence of TD and other basal ganglia-related conditions. A diagram illustrating the relationship between the microbiota, gut, and brain in TD has been recorded (157, 158). Changes in the microorganisms and surroundings can change the composition and functioning of the gut microbiome. These changes can cause an increase in the permeability of the gastrointestinal tract, allowing molecules from the gut to pass into the bloodstream and reach the brain. This may result in alterations in the functioning of the basal ganglia. The alteration of the gut microbiome can also lead to other potential hazards, including an uneven distribution of short-chain fatty acids, increased stimulation of the vagus nerve, and the release of substances produced by bacteria. A protein known as zonulin may have a function in controlling the permeability of the intestinal barrier (159). The presence of elevated zonulin levels indicates a compromised intestinal barrier, and measuring zonulin levels in the blood has been used as a way to measure intestinal permeability in neurological and mental disorders. The purpose of this is to gain a deeper understanding of how imbalances in gut bacteria and the strength of the gut barrier contribute to these diseases (160, 161). It is believed that if compounds from the intestine enter the peripheral blood vessels, we can study changes in their molecules using serum metabolomics. This can also help us understand how the microbiome in the intestine interacts with these metabolites, revealing new connections between changes in gut bacteria, metabolites in the blood, and physical disorders (162, 163).

Many investigations on the process of acupuncture treatment for TD are carried out through experiments using animal models. These experiments primarily concentrate on how acupuncture affects neurotransmitters in the body (164). There is insufficient study on how acupuncture operates in real-world medical practices. However, recent studies have begun to utilize omics analyses to gain insight into this mechanism. A new study using gut microbiome sequencing has revealed that acupuncture has the potential to modify gut microbiome structure, reduce inflammation in the central nervous system, strengthen gut barrier function, and regulate neurotransmitter levels in neuropsychiatric conditions (165–168). Regarding metabolomics tests, acupuncture may have the ability to treat neuropsychiatric conditions through the modulation of widespread inflammation and enhancement of lipid metabolism (169–171). QHN therapy has shown promise in effectively addressing TD in a clinical environment and merits further exploration to gain a deeper understanding of its underlying mechanisms. Currently, there is limited research on the application of acupuncture as a treatment for TD in the omics field (158, 172).

For a 12-week period, the authors examined the efficacy of acupuncture as a treatment for TS in children (8). The research involved 250 individuals who had been identified as having TS. Out of the total patients, 122 were treated with a combination of acupuncture and conventional treatment (observation group), while 128 patients solely underwent conventional treatment (control group). To account for variances in initial characteristics, propensity score matching was applied, leading to a sample of 78 individuals in each group. At the end of a 12-week treatment period, the overall score on the YGTSS was examined for improvement in both study groups. The findings indicate that both groups had comparable demographics and YGTSS scores after being matched. Significant enhancement in the YGTSS score was noted in the group being observed, in contrast to the control group. The contrast was particularly evident in individuals who had vocal tics. The results showed that acupuncture treatment was considerably more effective than traditional treatment in a clinical setting. According to the results, acupuncture could potentially be a helpful additional treatment for TD in children, particularly for vocal tics (8). In their research, Lu and colleagues performed a thorough evaluation of the efficacy and safety of acupuncture as a treatment for TD in children. Their objective was to enhance comprehension of acupuncture’s role in TD treatment and the current supporting evidence (153). This analysis examined RCTs that pitted acupuncture therapy against medication therapy for the management of TD, in order to evaluate their respective efficacies. A comprehensive exploration of six digital databases was carried out, encompassing the time span from the inception of the database up until April 2020. The potential for prejudice in the literature that was included was evaluated with the assistance of the Cochrane Collaboration’s tool. Furthermore, the information gathered from the included literature was examined using Review Manager 5.3. This literature review consisted of 22 RCTs with 1,668 participants. The findings of the meta-analysis showed that acupuncture proved to be more successful compared to medication in terms of overall effectiveness, decrease in YGTSS, lower occurrence of negative effects, and decreased chances of recurring. In contrast to using drugs, utilizing acupuncture as the sole method of treatment demonstrated increased effectiveness in decreasing YGTSS scores, a decreased occurrence of negative effects, and a lower likelihood of the condition recurring for those with TD (153).

Tang and colleagues examined the variation in the treatment effectiveness for infantile TS by comparing the combined method of acupuncture and pingganjianpi decoction with haloperidol tablets (173). 47 children were randomly split into two groups, an observation group with 25 children and a control group with 22 children. During the study, individuals in the observation group were given acupuncture therapy on designated acupuncture sites, including LR 3 (Taichong), GV 20 (Baihui), CV 12 (Zhongwan), and ST 36 (Zusanli), for a duration of 30 min daily. They also had a 5-day pause after every 10 treatments. The participants in the study were also given Pinggan jianpi decoction. Conversely, the control group was administered haloperidol tablets twice a day for 30 days, beginning with a low dose of 0.05 mg/kg per day, in a total of 3 cycles. The YGTSS was employed to assess the length, occurrence, and seriousness of tics prior to and after intervention over the course of 30, 60, and 90 days. The two groups were compared in terms of both their effectiveness and negative responses. The observation group showed overall effectiveness rates of 40.0, 64.0, and 76.0% at 30, 60, and 90 days, while the control group had rates of 59.1, 68.2, and 77.3% at the same time intervals. After receiving treatment for 30 days, the control group exhibited superior outcomes compared to the observation group. However, there were no notable distinctions between the two groups at other stages throughout the study. Every measurement of the duration, rate, and seriousness of tics showed a significant decline at every stage following treatment in both sets. At the 30-day mark, the control group displayed more substantial decreases in all areas compared to the observation group. However, there were no notable discrepancies at any other intervals. The risk of negative responses was lower in the observed group. Overall, using a combination of acupuncture and medication had comparable effectiveness to haloperidol tablets for treating infantile TS, with fewer undesirable effects (173).

Xiang and colleagues conducted a study on the effectiveness of local acupuncture in treating TD (174). Both groups saw improvement in YGTSS motor tic scores, with high success rates of 90.4 and 84.2% in the acupuncture and western medication groups respectively, without any significant difference in statistics. Acupuncture showed a 100.0% success rate for TTD, compared to 83.3% for medical therapy, suggesting acupuncture may be more effective for TTD. The success rates for TTD, CTD, and TS in the acupuncture group were 100.0, 88.2, and 84.2% respectively, with better results for TTD compared to CTD and TS. Overall, the effectiveness of acupuncture and medical therapy on TD was similar, but acupuncture showed superior effectiveness for TTD and potentially for preventing CTD or TS. Therefore, early intervention with acupuncture may achieve satisfactory results and potentially prevent the development of CTD or TS (174).

The researchers Zhu et al., investigated how various lengths of time that acupuncture needles were retained on scalp acupoints affected the treatment of TS (175). A total of 62 individuals with TS were randomly divided into an observation group and a control group, with 31 participants in each group. The observation group received 2 h of needle retention while the control group received 30 min. Specific acupoints, including the middle line of the forehead, middle line of the vertex, lateral line 1 of the vertex, anterior oblique line of the vertex-temporal, and posterior temporal line, were chosen as main acupoints, and were combined with adjuvant acupoints. Treatment was administered every other day for a period of 2 months. YGTSS and Tourette Syndrome Global Scale (TSGS) were used to evaluate the effectiveness of the treatment, and the results were then compared. Both groups showed significant improvement in kinetic and vocal tics after treatment, as indicated by a highly significant decrease in YGTSS and TSGS scores (*p* < 0.01). The overall success rate was 61.3% for the observation group and 67.7% for the control group, with no significant difference between the two groups. These findings suggest that both 2 h and 30 min of needle retention in scalp acupuncture therapy can effectively alleviate symptoms of TS, with similar therapeutic effects (175).

In a network analysis conducted by Chen and colleagues, they analyzed 86 studies to identify common acupoints used for treatment of TD through acupuncture techniques. Bai-hui (DU20), Feng-chi (GB20), LR3, Neiguan (PC6), He-gu (LI4), and San-yin-jiao (SP6) are the most common acupoints used for treating TDs. Combination of these points, especially Bai-hui (DU20), Neiguan (PC6) and SP6 could increase the efficacy of acupuncture for TD significantly (176).

Li and colleagues believed that twitching symptoms developed during TD progression are due to the injuries of tendons (177). Yang qi nourish tendons and the Governor Vessel is the master of the yang vessel. Thus, for the proper function of tendons and muscles, pushing them through Governor vessel and further yang qi is necessary (176). Governor Vessel is one of the most common meridians used for treatment of TD as it passes through posterior midline of the human body and connects to zang-fu organs as well as regulates yin and yang qi and blood of the whole body (176, 177).

There has been a significant increase in attention towards non-drug treatments for TD, and acupuncture has become a prominent contender in the field of alternative therapies. Acupuncture is highly regarded for its low risk of side effects and focus on overall well-being, as it works by targeting specific points on the body to improve neurological function and relieve symptoms. Recent scientific studies have highlighted its potential, with notable decreases in both the frequency and severity of tics reported. For instance, a clinical trial conducted by Qi and colleagues demonstrated that acupuncture was more effective than traditional treatments in providing relief for patients with TS (178). This ending is in line with more extensive research, including systematic reviews like those by Pu and others, which collectively confirm that acupuncture is effective in reducing tic symptoms in different groups (112).

However, unlike pharmacological treatments, Cognitive Behavioral Intervention for Tics (CBIT) is a non-medical approach that has been widely accepted and incorporated into the treatment of TD. By teaching patients alternative responses to tics, numerous studies have confirmed the efficacy of CBIT, including a significant randomized controlled trial conducted by Piacentini et al., which solidifies its effectiveness in reducing tic severity without the use of medication (179). In addition, recent approaches such as biofeedback have demonstrated potential in assisting patients to regulate physiological responses associated with their tic triggers. Research conducted by Nagai et al. in 2014 revealed that biofeedback significantly decreased tic symptoms by improving patients’ understanding and management of their bodily reactions (180). Both treatments, though equally successful, present unique approaches in contrast to acupuncture, providing numerous options for adapting patient treatment based on individual requirements and treatment outcomes.

As discussed before, TD has association with different comorbidities such as OCD, anxiety disorder, and ADHD. Recent studies suggested that acupuncture could be used for treatment of these comorbidities. Feng et al. showed that Transcutaneous electrical acupoint stimulation could be an effective treatment in addition to CBT and clomipramine as well as reduction of adverse effects of clomipramine (181). Amorim and colleagues found that acupuncture could be an effective treatment for anxiety disorders as it has more efficacy and less adverse effects compared to conventional treatments (46). However, there is a limited evidence for treatment of ADHD by acupuncture (46).

## 9 Discussion of limitations and future research directions

Although acupuncture has shown promise as a treatment for TD, there are several limitations that need to be taken into account. One major limitation is the concentration on short-term effects in current research. The long-term effectiveness and potential adverse effects of acupuncture have yet to be sufficiently investigated, which highlights the need for longitudinal studies. Furthermore, the variance in acupuncture methods used in different studies presents a challenge. The lack of standardization in techniques, selection of acupuncture points, and frequency of treatments makes it difficult to accurately compare and combine findings from various research studies. In addition, a significant portion of the current studies heavily prioritize quantitative measures such as reducing symptoms, thereby neglecting qualitative factors including patient contentment and mental wellness. These qualitative findings are essential, particularly in chronic illnesses such as TD, where improving quality of life is just as important as managing symptoms. Furthermore, the majority of studies have limited participant diversity and are confined to specific regions, limiting the applicability of their results to the wider global population with TD. Additionally, the use of traditional clinical evaluations to assess tic severity may not fully capture the overall effect of acupuncture on patients’ well-being. In the future, it is crucial that research focuses on creating long-term studies to evaluate the continued success and safety of using acupuncture to treat TD. Standardizing acupuncture procedures within clinical trials would enable better comparisons and establish definitive treatment recommendations. Utilizing qualitative research techniques would provide a more comprehensive understanding by capturing the wider effects of acupuncture treatment from the patient’s point of view. Broadening the scope of study participants to include varying ethnicities and age groups would increase the overall relevance and applicability of the results. Moreover, it is crucial to develop revolutionary evaluation methods that are specifically designed to evaluate the effects of acupuncture on TD. These methods can offer a more comprehensive understanding of how acupuncture influences the frequency and severity of tic symptoms. Additionally, conducting comparative studies that pit acupuncture against other conventional or complementary treatments could establish acupuncture as a viable therapeutic choice, allowing for a better understanding of its efficacy and potential integration into standard care protocols. After careful consideration, it is evident that while the initial evidence of acupuncture’s potential in managing TD is positive, it is imperative to make a collective effort to bridge the gaps in research. Doing so could greatly progress our comprehension of its efficacy and establish its rightful place in wider therapeutic approaches. Such progress is crucial not only for improving patient outcomes, but also for gaining the recognition and validation of acupuncture as a valuable treatment method in the medical field.

## 10 Conclusion

The increasingly popular use of herbal remedies for chronic diseases is influenced by personal values, beliefs, and health philosophies. Many people find that herbal remedies align better with their ideals than traditional Western medicine. Additionally, while chemical drugs are often prescribed for mental disorders, their side effects and limited effectiveness make alternative treatments, such as herbs, more appealing. The growing demand for herbal medicines highlights the need for scientifically validated herbal extracts and active ingredients. The use of “phytochemicals” could potentially provide a new source of beneficial neuroleptics. Acupuncture has been a key factor in promoting the acceptance of Chinese medicine globally. Its effectiveness and unique biological effects have made it a widely accepted therapy, even within the scientific community. Previous research on acupuncture anesthesia and acupuncture analgesia has greatly contributed to the understanding of pain physiology. As mentioned before, studies found that acupuncture could be a useful tool for not only the treatment of TD, but also its comorbidities such as anxiety disorder and OCD which suggested its utility as an efficient treatment against TD. The field of acupuncture research has rapidly developed in recent years, particularly with the advancement of biological technologies, such as functional genomics and proteomics, which have helped uncover the mechanism behind acupuncture’s effects. Acupuncture has been found to produce both non-specific and specific effects, mediated by various biomolecules. Non-specific effects, such as the release of endogenous opioids and purines, are produced by the acupuncture itself and are not dependent on a specific body state. On the other hand, specific effects, such as the action of biomolecules like S100A9 and CC10, are related to specific organ dysfunctions in disease states. By studying these biomolecules, further specific effective molecules could be discovered and used in the development of biological medicines. The emerging field of translational medicine, which combines research with application, offers great potential for the advancement of acupuncture and its corresponding drug discovery. As translational medicine becomes increasingly interdisciplinary, the integration of acupuncture effectiveness and target drug discovery will further promote the application and development of biological medicine.
